# Exploring the Influence of Insect Honeydew on Plant Physiology and Health: Bridging the Gap in Current Understanding

**DOI:** 10.1111/ppl.70623

**Published:** 2025-11-06

**Authors:** Jamin Ali, Rizhao Chen, Mohammad Danish, Riyazuddin Riyazuddin, Tarek Dardouri, Abdullahi Ahmed Yusuf, Eric Conti, Ahmet Bayram

**Affiliations:** ^1^ College of Plant Protection, Jilin Agricultural University Changchun China; ^2^ Department of Plant Physiology Comenius University Bratislava Slovakia; ^3^ Instituto de Bioingeniería, Universidad Miguel Hernández Elche Spain; ^4^ Agirodor Biopôle Rennes France; ^5^ Department of Zoology and Entomology University of Pretoria Pretoria South Africa; ^6^ Department of Agricultural, Food and Environmental Sciences University of Perugia Perugia Italy; ^7^ Department of Plant Protection, Faculty of Agriculture Dicle University Diyarbakir Türkiye

**Keywords:** honeydew composition, honeydew‐plant interactions, plant defense, plant disease, plant health, soil fertility

## Abstract

Honeydew, a sugary excretion from sap‐feeding insects, significantly influences plant‐insect interactions. While extensive research has examined honeydew's composition, regulation, and role in insect‐plant relationships, its direct effects on plant physiology and health remain understudied. This review synthesizes current knowledge about the effects of insect honeydew deposition on plant physiology and health, focusing on both beneficial and detrimental effects. Adverse impacts include sooty mold development, increased pathogen susceptibility, and reduced photosynthetic capacity due to surface occlusion. Beneficial effects encompass enhanced flowering, strengthened direct and indirect plant defense mechanisms, and improved soil fertility through indirect pathways. This review examines the relationship between honeydew composition and its effects on plants, while highlighting critical knowledge gaps in molecular mechanisms and long‐term plant responses. This synthesis based on a comprehensive literature search provides a foundation for future research on honeydew's role in plant health and ecological interactions.

## 1 Introduction

Studies on insect–plant interactions have traditionally focused on key topics such as insect feeding, oviposition, and their effects on plants, as well as plant responses aimed at avoiding insect herbivory through diverse defense strategies (Schoonhoven et al. 2005). When insects feed or oviposit, they transfer a variety of molecular patterns, serving as herbivore‐associated signals that modulate plant responses to insect herbivores (Rondoni et al. 2018; Erb and Reymond 2019; Mostafa et al. 2022; Hu et al. 2024). However, sap‐sucking herbivores, like aphids, whiteflies, scale insects, mealybugs, planthoppers, and leafhoppers (Hemiptera: Sternorrhyncha and several Auchenorrhyncha), not only damage plants directly through feeding punctures with injection of saliva and indirectly by transmitting plant viruses (Whitfield et al. 2015), but they also influence plant ecology, physiology and health through the release of honeydew (Nelson and Mooney 2022; de Bobadilla et al. 2024).

**FIGURE 1:**
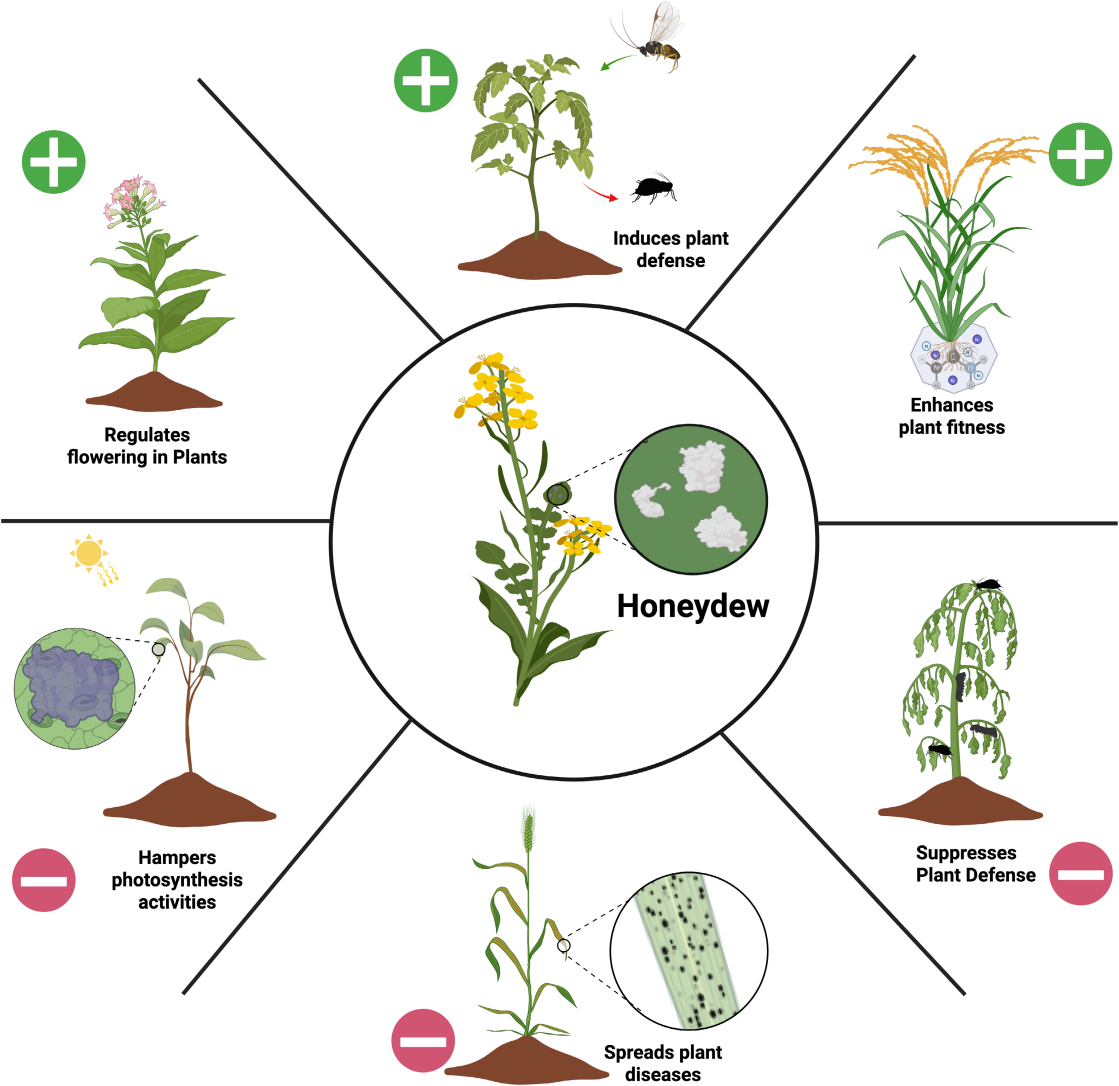
Illustration showing honeydew–plant interactions. Positive effects include regulation of flowering, induction of plant defenses, and enhanced plant fitness. Negative effects include reduced photosynthesis, spread of plant diseases, and suppression of defenses. Green (+) marks positive effects, while red (−) marks negative effects.

Honeydew, a sugary excretion excreted via the anal opening by sap‐sucking insects, interacts with plants in various ways, exhibiting both negative and positive effects (Figure 1). Often overlooked, honeydew acts as an important substance, influencing plant health and affecting responses between insects and plants. The composition and quantity of honeydew are influenced by various ecological factors, including the insect species involved, the plant species involved, the nutritional quality of plant sap, and environmental conditions (Fischer and Shingleton 2001; Schillewaert, Vantaux, et al. 2017; Blanchard et al. 2022). Beyond its role as waste, honeydew has ecological importance that extends to various aspects of the ecosystem. This sticky substance serves as a nutrient source for many organisms, including parasitic Hymenoptera (Buitenhuis et al. 2004; Tena et al. 2016; van Neerbos et al. 2020; Colazza et al. 2023), insect predators (Rondoni et al. 2018), ants (Nelson and Mooney 2022) and microorganisms (Álvarez‐Pérez et al. 2023). The microbial communities thriving in honeydew, in turn, contribute to the broader ecological landscape (van Neerbos et al. 2020; Colazza et al. 2023). Recognizing honeydew as a vital substance with a significant impact on plant physiology, including reproduction and health, opens the door to understanding its complex contributions to ecological processes (Owen and Wiegert 1976; Tena et al. 2016; Álvarez‐Pérez et al. 2023). Additionally, the excretion of honeydew droplets gives the plant a chance to recognize insect herbivores (VanDoorn et al. 2015). Thus, honeydew represents another potential source of herbivore‐associated molecular patterns (HAMPs); however, this area has not been studied in detail yet (Wari et al. 2019). This review highlights the complex relationship between honeydew and plant physiology, emphasizing its crucial role in shaping plant health. While existing research mainly focuses on honeydew composition, regulation, microbial communities, and its influence on insect‐plant interactions, the connection between honeydew and plant health, as well as its direct impact on plant physiology, remains largely unexplored.

Exploring the dual impact of honeydew on plant physiology, we evaluate both its negative and positive aspects. A negative effect of honeydew interactions with plants is that it creates an environment conducive to microbial pathogens, potentially escalating instances of plant health issues (Figure 1) (Wari et al. 2019). Moreover, the deposition of honeydew and consequent development of fungi (sooty molds) on leaf surfaces holds the potential to impede photosynthetic activities due to surface coverage (Nelson 2008; Chomnunti et al. 2014). However, honeydew also plays a role in plant defenses, exerting both suppressive and inductive effects (Schwartzberg and Tumlinson 2014). Additionally, it has been linked to flowering regulation, though this remains largely unexplored (Cleland 1974). Studies further suggest that honeydew can enhance soil fertility, contributing to improved plant health (Owen and Wiegert 1976; Buckley 1987).

Moreover, studies explore the release of volatile compounds from honeydew and its kairomonal activities, as well as its significance in recruiting biocontrol agents and managing pests (Leroy et al. 2014). This focus highlights a key reason why the direct effects of honeydew on plant physiology remain an underexplored field: a traditional division in research priorities. Research driven by entomological interests has naturally focused on honeydew's role in tritrophic interactions and indirect plant defense (e.g., predator/parasitoid recruitment). Conversely, research centered on plant responses to biotic stress has primarily investigated direct defense signaling or the impacts of pathogens, often overlooking the unique physiological consequences of a sustained, insect‐derived metabolic deposit like honeydew. This disciplinary divide has left a critical gap in understanding. Consequently, while the effects of honeydew on plant defense have been partially explored, its impact on other physiological aspects of plants remains poorly understood (Leroy et al. 2011; Schwartzberg and Tumlinson 2014; Wari et al. 2019). A limited number of studies from almost five decades ago briefly touched upon honeydew's role in plant flowering, with no subsequent research in that direction (Cleland 1974; Cleland and Ajami 1974). Similarly, poorly explored topics include honeydew's potential contributions to plant disease, its role in enhancing secondary infections through the proliferation of microbial pathogens, the consequences of its deposition on leaf surfaces, and its effects on the photosynthetic activity of plants. Here, we highlight the least explored areas, pointing out the gaps in our current understanding and suggesting potential directions for future research and practical applications of honeydew in ecological practices. These include strategies like integrated pest management, where honeydew could support biological control agents, as well as enhancing pollination and promoting biodiversity conservation through the attraction of beneficial insects.

In this review, we focus on a structured analysis, examining the complex interaction between honeydew composition, regulatory mechanisms, and plant‐related factors. We investigate the detrimental effects of honeydew deposition on plant surfaces, such as challenges posed by the proliferation of sooty molds and microbes, ultimately affecting photosynthetic activities by covering the plant surface. We also explore the consequences of the honeydew‐plant relationship in agricultural settings. Our exploration of the positive effects of honeydew on plants includes improved flowering, strengthened direct and indirect defense mechanisms, and an indirect yet important impact on soil fertility, boosting overall plant health. This review synthesizes current knowledge by evaluating both the detrimental and beneficial aspects of honeydew, the key themes of which are summarized in Table 1, before delving into a detailed analysis of its composition, mechanisms, and agricultural implications.

**TABLE 1:** A comprehensive overview of honeydew's dual effects on plant physiology and health.

Category of effect	Specific effect/phenomenon	Mechanism/key findings	Consequence for plant health	References
Negative effects				
Photosynthesis and pollination	Reduction in photosynthetic rate	Physical occlusion of stomata and leaf surfaces by honeydew and subsequent sooty mold growth, hindering gas exchange and light interception.	Decreased carbon assimilation, reduced plant growth and productivity, accelerated leaf senescence.	(Rabbinge et al. 1981; Nelson 2008; Chomnunti et al. 2014)
	Disruption of pollination	Coating of reproductive structures (e.g., maize tassels/silks) with honeydew, making them sticky and dysfunctional.	Impaired pollen transfer, yield losses, and contamination of marketable produce.	(Carena and Glogoza 2004; Edde 2022)
Pathogen promotion	Enhanced fungal pathogen growth	Honeydew acts as a nutrient‐rich medium, stimulating spore germination (e.g., *Cochliobolus sativus*, *Septoria nodorum*) and germ‐tube growth.	Increased susceptibility to necrotrophic fungal infections and greater disease severity.	(Fokkema et al. 1983)
	Altered phyllosphere microbiome	Promotes rapid colonization of saprophytic fungi (e.g., *Aureobasidium pullulans*) and can shift microbial community dynamics.	Can initially stimulate saprophytic fungi that compete with pathogens, but often primes the leaf surface for secondary infections by necrotrophic fungi.	(Fokkema et al. 1983)
	Reduced fungicide efficacy	Honeydew interferes with chemical activity, and fungicide suppression of saprophytes reduces biological competition against pathogens.	Lowered effectiveness of chemical control measures, leading to poorer disease management.	(Dik and Van Pelt 1992; Müllenborn et al. 2008)
Defense suppression	Phytohormonal crosstalk (SA‐JA)	SA present in aphid honeydew is taken up by the plant, inducing the SA pathway and cross‐inhibiting the JA pathway.	Impairs the plant's ability to mount effective defenses against chewing herbivores and necrotrophic pathogens.	(Schwartzberg and Tumlinson 2014; VanDoorn et al. 2015)
Positive effects				
Flowering regulation	Bioactivation of salicylic acid (SA)	Aphids ingest inactive SA‐glycosides from phloem, hydrolyze them in the gut, and excrete bioactive SA in honeydew. This free SA can induce flowering in receiver plants.	Induces flowering in specific species (e.g., *Lemna gibba* ) under non‐inductive conditions, potentially synchronizing reproduction.	(Cleland 1974; Cleland and Ajami 1974)
Defense induction	Direct and indirect defense elicitation	Honeydew contains Herbivore‐Associated Molecular Patterns (HAMPs) that trigger defense signaling (Ca^2+^, ROS, MAPKs), leading to phytoalexin accumulation and defense gene (e.g., *PR1*, *PR10*) activation.	Enhanced resistance against subsequent pest or pathogen attacks.	(VanDoorn et al. 2015; Alamgir et al. 2016; Wari et al. 2019; Zhu et al. 2020)
		Emission of specific Volatile Organic Compounds (VOCs) induced by honeydew deposition.	Attracts natural enemies (parasitoids and predators) of the herbivores, facilitating indirect defense.	(Wari et al. 2019)
	Pest detection	Honeydew serves as a detection cue for plants, allowing them to identify the presence of low‐damage herbivores like whiteflies.	Enables earlier and more targeted activation of defense responses.	(VanDoorn et al. 2015)
Indirect and ecosystem benefits	Recruitment of natural enemies	Honeydew serves as a direct food source (sugars, amino acids) for parasitoids and predators.	Strengthens top‐down biological control, reducing pest populations and plant damage.	(Wäckers et al. 2008; Leroy et al. 2011; Li et al. 2025)
		Microbes in honeydew produce kairomonal volatiles (e.g., DL‐lactic acid) that attract natural enemies.	Enhances the “recruitment” efficacy of biological control agents to infested plants.	
	Support for pollinators	Honeydew is used as a carbohydrate source by bees and other pollinators.	Increases pollinator activity in the area, which may improve pollination services for co‐flowering plants.	(Wäckers et al. 2008)
Soil fertility and biogeochemistry	Altered soil nutrient cycling	Large quantities of honeydew (e.g., 1 kg m^−2^ yr.^−1^ under lime trees) add dissolved organic carbon (DOC) to the soil, stimulating microbial biomass and activity.	Increases soil respiration and organic matter decomposition. Can lead to nitrogen immobilization by microbes, potentially reducing short‐term plant‐available N.	(Llewellyn 1972; Stadler and Michalzik 1998; Michalzik and Stadler 2005)
		Honeydew deposition changes throughfall chemistry, altering the fluxes of DOC, DON, NO_3_^−^, and NH_4_^+^.	Creates a complex interplay between carbon enrichment and nitrogen dynamics, with context‐dependent outcomes for plant fitness.	(Stadler and Michalzik 1998; Seeger and Filser 2008)

## 2 Methodology

To ensure a comprehensive and unbiased coverage of the topic, a systematic search of the literature was conducted using the PRISMA approach (Page et al. 2021) across the academic databases Web of Science Core Collection (WoS) (1980–2025), Scopus, and JSTOR. This search included articles, reviews, proceeding papers, and book chapters. Supplemental searches were performed using Google Scholar to identify additional relevant articles and gray literature. The search strategy employed key terms and Boolean operators in the title such as: (“honeydew”) by excluding non‐relevant web of science categories (Physics, Medicine etc.), which yielded in 490 results. Then within these results, they were sub‐categorized using the following keywords: (honeydew AND “plant*”) OR (“aphid” “whitefly” OR “health” OR “defense” OR “defense” OR “flowering” OR “soil fertility” OR “photosynthesis” OR “pathogen” OR “fungi” OR “bacteria” OR “microorganism” OR “Sternorrhyncha” OR “leafhoppers” OR “planthoppers” OR “mealybugs” OR “scale insects”). The reference lists of key articles were also screened for relevance. Papers published before 1980, which were unsearchable in Web of Science due to its time coverage restriction, were searched using Google Scholar with the same search terms. These papers were then obtained from JSTOR or other online sources. Inclusion criteria after carefully screening, focused on studies that investigated a direct physiological, biochemical, or plant health response upon the deposition of insect‐produced honeydew. Exclusion criteria omitted studies that focused solely on honeydew composition without linking it to plant responses, or those investigating honeydew only in the context of tritrophic interactions without measuring plant parameters. The findings from the selected literature were then synthesized into a narrative format, structured around key thematic areas: the negative impacts of honeydew (e.g., on photosynthesis and pathogen promotion), its positive effects (e.g., on defense induction and soil ecology), and its agricultural implications.

## 3 Origin and Composition of Honeydew

Honeydew is a sugary excretion produced by various hemipteran insects, including aphids (Aphididae), whiteflies (Aleyrodidae), mealybugs (Pseudococcidae), scale insects (Coccidae), psyllids (Pysillidae), leafhoppers (Cicadellidae), treehoppers (Membracidae) and froghoppers (Cercopidae) (Santas 1989; A. E. Douglas 2009; VanDoorn et al. 2015; Van Emden and Harrington 2017; Nelson and Mooney 2022). These insects feed on plant sap using their specialized mouthparts to pierce plant tissues and extract phloem and/or xylem sap. While most honeydew‐excreting insects primarily feed on phloem (suborder Sternorrhyncha), leafhoppers, treehoppers, and froghoppers (suborder Auchenorrhyncha) are mainly xylem sap feeders (Lt and Nault and Rodriguez 1985; Byrne and Bellows Jr 1991; Dietrich 2009; Hodkinson 2009; Weintraub 2009; Van Emden and Harrington 2017; Nelson and Mooney 2022; de Bobadilla et al. 2024).

Phloem sap is rich in sugars and other nutrients but lacks sufficient amino acids for the insects' needs. After ingestion, the sap undergoes processing within the insect's digestive system. Surplus sugars and other non‐essential components are then excreted as honeydew (waste). This process allows the insects to concentrate the essential amino acids and nutrients while eliminating excess sugars. Honeydew may also contain secondary plant compounds or even unmetabolized residues of systemic insecticides, depending on the insect's diet and environment (Molyneux et al. 1990; Fischer and Shingleton 2001; Tena et al. 2016; Calvo‐Agudo et al. 2020; Quesada et al. 2020; Shaaban et al. 2020; Starr 2021; Álvarez‐Pérez et al. 2023). In addition to sugars, honeydew contains a range of amino acids and various proteins from both the insect host and its microbiota (Molyneux et al. 1990; A. E. Douglas 2009; Dhami et al. 2011; Sabri et al. 2013). For example, the honeydew of the pineapple mealybug ( *Dysmicoccus brevipes* ) comprises up to 98% carbohydrates, including 55% cane sugar, 25% invert sugar, and 13.9% dextrin, along with amino acids (Auclair 1963; Dhami et al. 2011; Shaaban et al. 2020).

The sugars found in honeydew, such as melezitose, erlose, raffinose, trehalose, and trehalulose, are produced through the action of gut enzymes derived from plants within insects (Hendrix et al.). The composition and quantity of sugars present in honeydew can vary depending upon the insect species, the host plants, plant–root microbiome interactions, host plant infection by pathogens, and environmental conditions (Table 2) (Fischer and Shingleton 2001; Fischer et al. 2002; Hogervorst et al. 2007; Tena et al. 2013; Hijaz et al. 2016; Blanchard et al. 2022). For instance, aphids like *Metopeurum fuscoviride* and *Cinara* spp. (Hemiptera: Aphididae) produce honeydew rich in melezitose, while others like *Macrosiphoniella tanacetaria* and *Macrosiphum euphorbiae* produce minimal amounts of this sugar (Hendrix et al. 1992; Völkl et al. 1999). The age of aphids significantly influences honeydew production, with sugar concentration remaining stable while amino acid levels increase as the aphids age (Fischer et al. 2002). While the precise molecular mechanism for this shift remains unconfirmed, it is consistent with the fundamental life‐history strategy of aphids: mature, reproductive adults channel a greater proportion of ingested nitrogen toward egg production (A. E. Douglas 2003; Wilkinson and Douglas 2003). This likely increases the metabolic processing of phloem sap to meet the high demand for essential amino acids, resulting in their relative increase in excreted honeydew.

**TABLE 2:** Key factors influencing the composition and production of insect honeydew.

Factor	Host plant(s)	Insect species	Key effect on honeydew	References
Insect and plant species	*Populus tremula, P* *. alba*	*Chaitophorus populialbae, C. populeti*	High production of the trisaccharide melezitose.	(Fischer and Shingleton 2001)
	*Gossypium hirsutum, Euphorbia pulcherrima, Lycopersicon esculentum*	*Bemisia tabaci, Trialeurodes abutilonea, T. vaporariorum*	High proportion of oligosaccharides and a high concentration of turanose.	(Hendrix et al. 1992)
	*Tanacetum vulgare, Vicia faba, Chenopodium album*	*Aphis fabae*	Predominantly characterized by high levels of melezitose.	(Fischer et al. 2005)
Phloem sap variability	*V. faba*	*A. fabae*	Alterations in melezitose and other carbohydrate levels are directly dependent on the host plant.	(Schillewaert, Parmentier, et al. 2017; Schillewaert, Vantaux, et al. 2017)
Insect microbiota	*V. faba*	*Acyrthosiphon pisum*	Honeydew contains a complex mix of proteins from the aphid, its gut flora, and endosymbiotic bacteria (e.g., GroEL, flagellin).	(Sabri et al. 2013)
Insect species and geography	*Nothofagus solandri, N* *. truncata* , *N* *. fusca*	*Ultracoelostoma* spp., *Coelostomidia wairoensis*	Scale insect species and their local environment significantly impact honeydew chemistry, shaping consumer communities.	(Dhami et al. 2011)
Climate (temperature/CO_2_)	*V. faba*	*A. fabae*	Elevated temperature and CO_2_ significantly increase fructose concentration, with minor increases in honeydew volume and melezitose.	(Blanchard et al. 2022)
Aphid age	*T. vulgare*	*Metopeurum fuscoviride*	Sugar composition remains stable, but concentrations of specific amino acids (asparagine, glutamine) increase with aphid age.	(Fischer et al. 2002)
Diurnal rhythm	*Solanum tuberosum*	*Macrosiphum euphorbiae, Myzus persicae*	Honeydew production is 1.9 to 2.6 times higher during daylight. The sucrose‐to‐amino acid ratio and amino acid composition also shift.	(Taylor et al. 2012)
Ant attendance	Various	Various aphid species	Ant‐tended aphids produce honeydew richer in melezitose, while untended aphids produce honeydew with higher glucose content.	(Fischer and Shingleton 2001)

Honeydew composition varies not only among different insect species on the same host plant but also among different host plants for the same insect species (Schillewaert, Parmentier, et al. 2017; Schillewaert, Vantaux, et al. 2017). The production of honeydew is a complex process and its quantity is influenced by various factors. Certain species release more honeydew than their own body weight on an hourly basis, and this process is affected by factors such as insect age, size, species, seasonal and geographical location of the host plant, diurnal shifts, climate, plant–rhizobia interactions and host plant infection by pathogens (Hertel and Kunkel 1977; Hendrix et al. 1992; Douglas 1993, A. E. Douglas 2009; Fischer and Shingleton 2001; Fischer et al. 2002; Wool et al. 2006; Taylor et al. 2012; Whitaker et al. 2014; Hijaz et al. 2016; Blanchard et al. 2022). A comprehensive understanding of the factors influencing honeydew composition and quantity is crucial for elucidating the broader implications of its production, thereby illuminating the complex relationship between honeydew and plant biochemistry, and consequently, the resultant composition of honeydew (Fischer et al. 2005; A. E. Douglas 2009; Dhami et al. 2011; Hijaz et al. 2016). The influence of these factors on honeydew composition is supported by a range of experimental studies, as summarized in Table 2.

## 4 Negative Effects of Honeydew on Plants

Honeydew introduces negative physiological and ecological impacts on plants and can compromise their fitness. This section explores the adverse effects of honeydew on plants, categorizing them into distinct dimensions.

### 4.1 Impact on Photosynthetic Activity and Pollination

**FIGURE 2:**
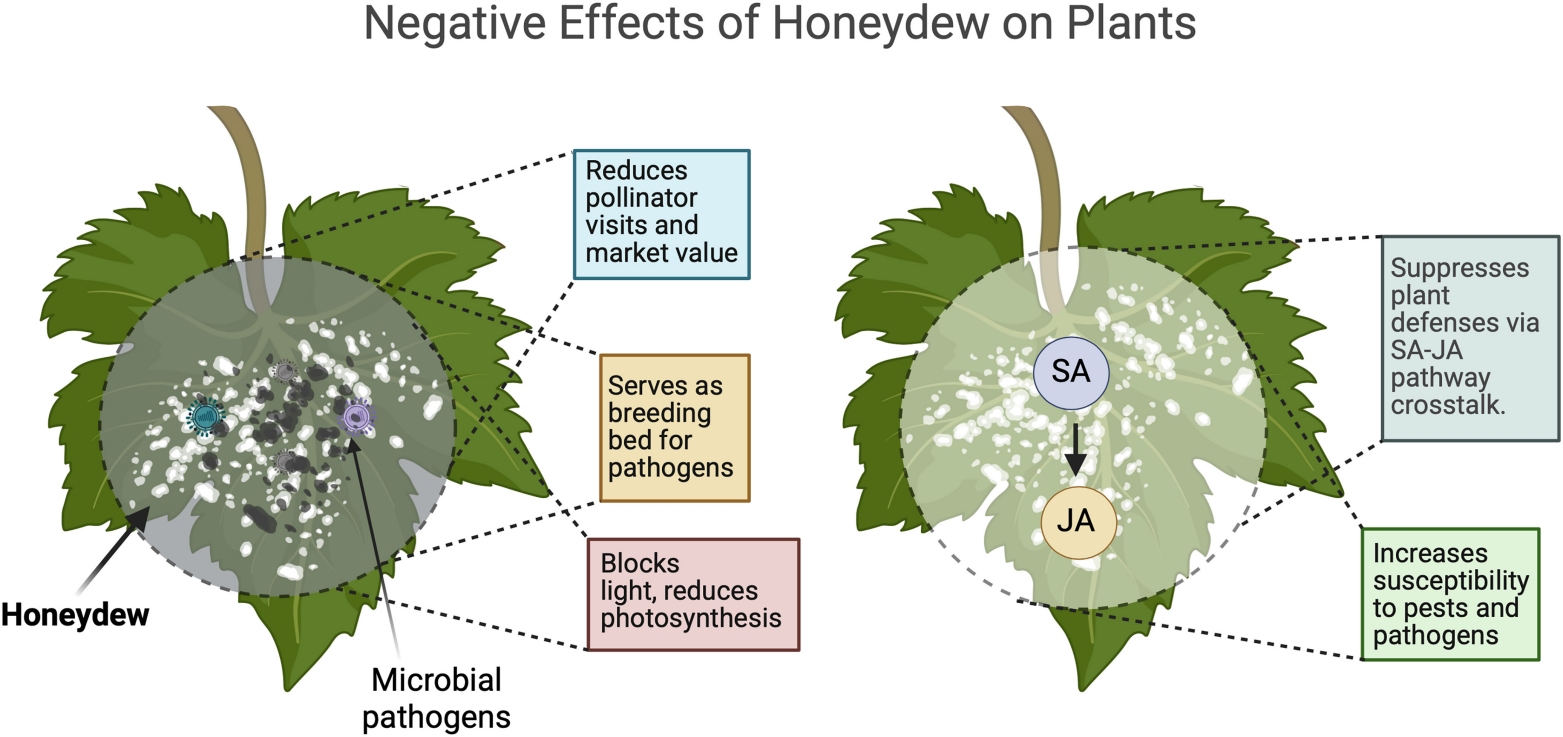
Honeydew deposition promotes microbial pathogen proliferation on plant surfaces by enhancing spore germination of necrotrophic fungi, stimulating saprophytic fungal colonization, reducing fungicide efficacy, and altering phyllosphere microbial communities to create conditions for secondary infections. Furthermore, honeydew can suppress plant defenses via crosstalk between salicylic acid and jasmonic acid (SA‐JA) pathways and reduce pollination by deterring pollinator visits. Honeydew composition may also reflect plant resistance, as some biochemical markers (e.g., cholesterol, β‐sitosterol) indicate resistant cultivars (Fokkema et al. 1983; Dik and Van Pelt 1992; Fujita et al. 2013; Shah et al. 2016).

The excretion of honeydew by hemipteran sap‐feeding insects poses a detrimental impact on plant physiology. The honeydew accumulation on foliage and stems provides a conducive environment for the growth of epiphytic fungi, particularly sooty molds, which represent a well‐known subgroup characterized by their dark mycelial mats on leaf surfaces. While other epiphytic fungi may also colonize honeydew deposits, sooty molds are especially relevant in this context because of their strong capacity to block light interception and interfere with photosynthesis (Crawford 1921; Nelson 2008; Chomnunti et al. 2014). Not only sooty molds but the presence of honeydew itself may impede the efficiency of photosynthetic activities, potentially leading to reduced plant productivity and growth (Figure 2). For instance, winter wheat experiences adverse effects due to honeydew application. A decrease in the maximum rates of photosynthesis in wheat flag leaves was observed both one and 7 days after honeydew application in a controlled environment (Rabbinge et al. 1981). The immediate reduction in photosynthesis was attributed to hindered gas exchange caused by stomatal clogging. For the long‐term impact, honeydew was linked to accelerated leaf senescence, negatively affecting leaf photosynthesis (Vereijken 1979; Rabbinge et al. 1981).

Honeydew promotes the growth of perthotrophic fungi, which form a layer on the leaf surface. This fungal layer interferes with the leaf's gas exchange processes, leading to a reduction in maximum photosynthesis rates (Dik 1990). In maize, heavily honeydew‐coated tassels and/or silks can lead to disrupted pollination, consequently causing yield losses. The occurrence of excessive honeydew on maize ears can make them visually unattractive and unsuitable for the market as also happens with other agricultural products (Carena and Glogoza 2004; Edde 2022). Moreover, the invasive mealybug *Pseudococcus comstocki* (Hemiptera: Pseudococcidae) exacerbates the situation by causing severe damage to apples, pears, and peaches in Italy through honeydew‐fuelled sooty mold outbreaks, adversely impacting agricultural productivity (Pellizzari et al. 2012).

### 4.2 Making Plant Surface Prone to Pathogens

Honeydew serves as a breeding ground for microbial pathogens, creating conditions that promote plant infections and diseases (Figure 2). Its deposition on plant surfaces is often linked to the growth of fungal pathogens and saprophytes on plant leaves (Fokkema et al. 1983). Beyond promoting pathogen growth, honeydew can also provide insights into plant resistance mechanisms. For instance, honeydew from plant hoppers feeding on resistant *Oryza* lines contained higher levels of cholesterol and beta‐sitosterol, which act as feeding deterrents, suggesting that honeydew composition can reflect biochemical differences between resistant and susceptible plants and influence pest feeding behavior (Fujita et al. 2013).

Aphid honeydew enhances wheat leaf infection by necrotrophic pathogens like *Septoria nodorum* and *Cochliobolus sativus*, increasing spore germination rates 2.5–4 times by stimulating the formation of multiple germ tubes per conidium and promoting overall germ‐tube growth during *C. sativus'* pre‐penetration phase (Fokkema et al. 1983). Its nutrient‐rich composition enhances fungal growth and aggressiveness by promoting cell wall‐degrading enzymes, facilitating the infection process. Conversely, the presence of aphid honeydew on wheat leaves significantly increases the colonization of various saprophytic fungi such as basidiomycetes *Sporobolomyces roseus, Cryptococcus laurentii var. flavescens* and the ascomycetes *Aureobasidium pullulans*, and *Cladosporium cladosporioides*, with their population densities increasing tenfold within about 6 days (Fokkema et al. 1983). While honeydew stimulates both saprophytic and pathogenic fungi, competition between these groups can influence plant health. In some cases, rapid saprophyte growth depletes nutrients, limiting pathogen proliferation and potentially reducing infection rates. However, in controlled experiments, honeydew's stimulatory effects on pathogens are more pronounced than in field conditions, likely due to complex microbial interactions on leaf surfaces (Fokkema et al. 1983).

Beyond promoting pathogen growth, honeydew reduces fungicide efficacy against necrotrophic pathogens in wheat (Rabbinge et al. 1984; Dik and Van Pelt 1992). Field studies indicate that honeydew interferes with fungicidal activity, particularly when broad‐spectrum fungicides suppress natural saprophytes. The suppressive effect of broad‐spectrum fungicides on saprophytic communities is well documented. For instance, in wheat, triazole fungicides (e.g., prothioconazole, tebuconazole) significantly inhibited saprophytic fungi such as *Alternaria alternata, Aspergillus niger, Rhizopus oryzae*, and *Trichoderma* spp., which are natural antagonists of pathogenic *Fusarium* species (Müllenborn et al. 2008). Strobilurins (e.g., azoxystrobin) were largely ineffective against Fusarium spp. but did affect certain saprophytes, notably *Microdochium majus*, which was highly sensitive (Müllenborn et al. 2008). This fungicide‐induced disruption of the saprophytic community reduces competitive pressure on pathogens and removes a vital layer of biological control, thereby facilitating the exploitation of honeydew‐rich environments by pathogens and contributing to the reduced efficacy of chemical treatments. This effect is especially pronounced in pathogens with short latent periods, such as *S. nodorum*, which rapidly exploit honeydew's nutrient availability (Dik and Van Pelt 1992). Unconsumed honeydew also serves as a substrate for soil microorganisms (Lazzari and Zonta‐de‐Carvalho 2012). In New Zealand, honeydew from the passion‐vine hopper (*Scolypopa australis*, Hemiptera: Ricaniidae) contributes to up to 85% of kiwifruit losses (Tomkins et al. 2000). Similarly, the date palm hopper ( *Ommatissus lybicus* , Hemiptera: Tropiduchidae) can cause up to 50% yield losses in date crops, with honeydew‐induced sooty mold further reducing photosynthesis and weakening plant health (Shah et al. 2016).

### 4.3 Suppression of Plant Defenses

The dynamic interaction between honeydew and plants encompasses various aspects, one of which is its negative impact on the plant's defense system (Figure 2). The composition of insect honeydew is remarkably complex, encompassing a diverse array of proteins from insects, plants, and microbes. These components have the potential to shape various interactions within plant–insect–microbe systems. Honeydew application on tomato plant leaves has been shown to alter phytohormonal signaling (VanDoorn et al. 2015). This discovery implies a potential mechanism through which honeydew may weaken plant defenses. The underlying reason for honeydew's suppression of plant defenses lies in the crosstalk between the salicylic acid (SA) and jasmonic acid (JA) pathways induced by aphid honeydew (Schwartzberg and Tumlinson 2014). Specifically, the study highlights how honeydew application leads to the inhibition of wound‐induced JA accumulation, thereby impairing the plant's ability to respond effectively to damage. This effect is primarily attributed to the presence of SA in aphid honeydew, which induces SA within the leaf tissue rather than accumulating from exogenous application (VanDoorn et al. 2015).

Additionally, honeydew contains biologically active constituents such as sugars and sugar conjugates, which may influence plant defense responses (Schwartzberg and Tumlinson 2014). Pea aphid honeydew is composed of various sugars, including fructose, glucose, sucrose, and trehalose, the latter of which has been implicated in regulating wound‐ and pathogen‐related gene expression (Bae et al. 2005; A. Douglas 2006). Furthermore, approximately half of the SA in honeydew exists in a conjugated form, which may contribute to SA accumulation in plants (Klick and Herrmann 1988). The presence of bacteria in honeydew may also play a role, as bacterial flagellin has been shown to induce systemic acquired resistance (SAR) in plants (Zipfel et al. 2004). Despite these important findings, this area remains understudied. Interestingly, the honeydew resulting from infection by the ergot fungus *Claviceps purpurea* (a microorganism from a different biological domain) contains pathogenesis‐related enzymes such as CatD, an extracellular catalase. This enzyme is thought to play a role in pathogenicity and suppression of plant defense mechanisms (Garre et al. 1998). Therefore, further research is needed to comprehensively understand the specific mechanisms and compounds not only associated with sucking insects but also with honeydew produced by other organisms (e.g., the ergot fungus *Claviceps purpurea*) that may suppress plant defense.

## 5 Positive Effects of Honeydew on Plants

Honeydew, while showing negative impacts on plants, also offers various benefits.

### 5.1 Regulation of Flowering in Plants

**FIGURE 3:**
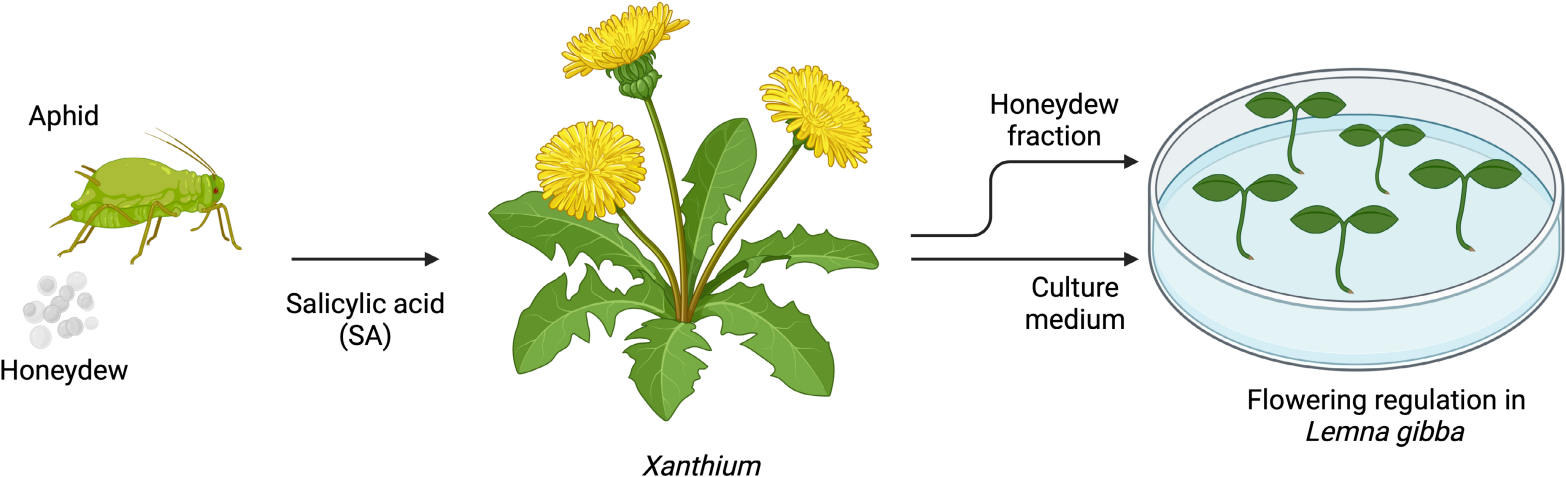
Honeydew‐mediated flowering regulation via salicylic acid (SA) bioactivation. Aphids feeding on *Xanthium* phloem ingest SA‐glycosides, which are hydrolysed in the aphid gut to free, bioactive SA and excreted in honeydew. When transferred to receiver plants such as *Lemna gibba* , this SA induces flowering under non‐inductive photoperiods by reducing vegetative frond production, triggering frond thickening, and initiating floral development. This effect is species‐specific, as SA‐responsive pathways are present in *Lemna* but not in *Xanthium* or *Spirodela* (Cleland 1974; Cleland and Ajami 1974).

Honeydew may influence flowering through the bioactivation and delivery of salicylic acid (SA), a key floral inducer. When aphids (*Dactynotus ambrosiae*) feed on *Xanthium strumarium* , they ingest SA glycosides, the bound, inactive form present in plant phloem (Figure 3) (Cleland 1974). During digestion, aphid glycosidases hydrolyse these glycosides, converting them into free, bioactive SA (Cleland and Ajami 1974). This free SA is excreted in honeydew and, when transferred to receiver plants like *Lemna gibba* , directly induces flowering under non‐inductive photoperiods at concentrations as low as 5.6 μM. The mechanism involves SA mimicking photoperiodic signals: it reduces vegetative frond production by 60%–70% while triggering frond thickening (“gibbosity”) and floral initiation, phenotypes identical to long‐day‐induced flowering (Cleland and Ajami 1974). Crucially, this effect is species‐specific; SA in honeydew induces flowering in *L. gibba* and *L. minor* but not in *Xanthium* or *Spirodela*, suggesting receiver plants require specific SA‐responsive pathways (Cleland 1974; Cleland and Ajami 1974). Thus, honeydew may function as a bioactive SA vector, linking aphid feeding physiology to developmental responses in neighboring plants.

### 5.2 Induction of Plant Defenses

**FIGURE 4:**
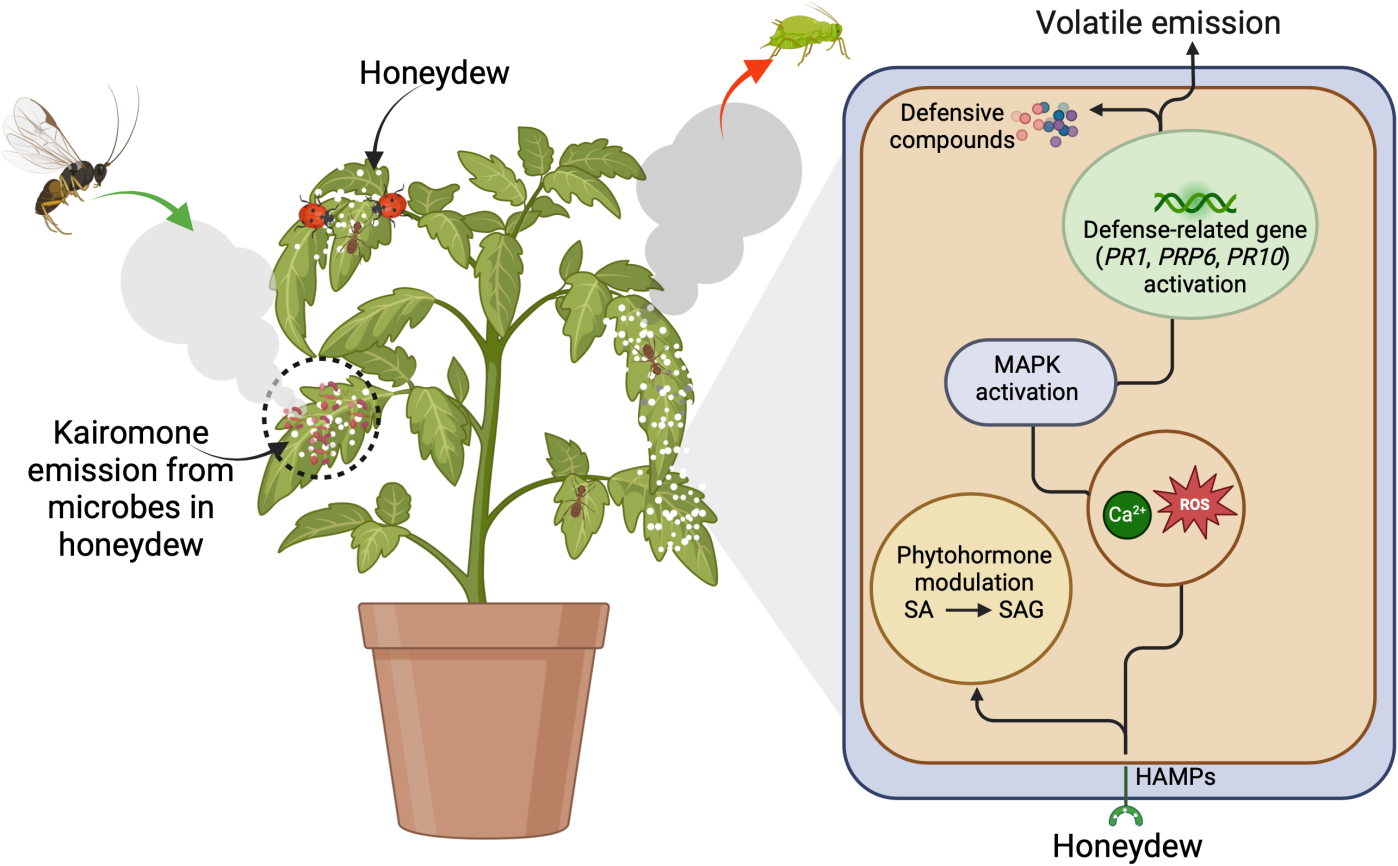
Honeydew deposition induces plant defenses through recognition of HAMPs, triggering intracellular signaling involving Ca^2+^, ROS, and MAP kinases. This leads to phytohormone modulation and activation of defense‐related genes, resulting in the accumulation of defensive compounds and emission of VOCs. Simultaneously, honeydew supports microbial communities that emit kairomones to attract natural enemies, provides nutrition for predators and parasitoids, and serves as a food source for pollinators. Arrows indicate directional interactions (Schwartzberg and Tumlinson 2014; VanDoorn et al. 2015; Wari et al. 2019; Li et al. 2025).

The dynamic interaction between honeydew and plants encompasses various aspects, one of which is its impact on the plant's defense system. This influence can be categorized into several dimensions. To begin with, honeydew has a manipulative role as a potent plant defense elicitor by inducing plant defenses primarily through converting SA levels into a less active glycoside form, salicylic acid glycoside (SAG), which can still induce a defense response without triggering an immediate strong reaction (Figure 4) (Schwartzberg and Tumlinson 2014). Moreover, honeydew excreted by the brown plant hopper (BPH) *Nilaparvata lugens* (Hemiptera: Delphacidae) was found to elicit both direct and indirect defenses in rice plants (Wari et al. 2019). This effect was attributed to the accumulation of phytoalexins in the leaves and the release of volatile organic compounds (VOCs). Additionally, it activates defense‐related genes (*PR1* and *PRP6*, *PR10*), prompting the synthesis of defensive compounds, including SA and phytoalexins (VanDoorn et al. 2015; Wari et al. 2019; Zhu et al. 2020). These compounds play a role in affecting insect physiology and performance on the plants (Alamgir et al. 2016).

Beyond these direct and indirect defense pathways, honeydew emerges as a key player in shaping early events in the rice defense cascade, significantly influencing phytohormone levels and potentially affecting downstream signal transduction mechanisms (Wasternack and Song 2017). The complex interaction between honeydew and plant defense extends beyond phytohormones, prompting a comprehensive exploration of additional signaling components. This investigation encompasses Ca^2+^‐mediated responses (Arimura et al. 2011; Zhu et al. 2020), levels of reactive oxygen species (ROS) (Zebelo and Maffei 2015; Shinya et al. 2016; Zhu et al. 2020), and the activity of mitogen‐activated protein (MAP) kinases (Hettenhausen et al. 2015). Furthermore, the role of honeydew‐induced transcription factors as crucial connectors, directly links signaling events to the activation of defense genes (Woldemariam et al. 2011; Wari et al. 2019). Honeydew deposition is also utilized by plants for the detection of insect herbivores. For instance, some insects, such as whiteflies, feed on plants without causing considerable damage to mesophyll cells, making it difficult for plants to detect them. However, honeydew deposition by whiteflies on plants aids in the identification of the pest due to the presence of whitefly‐associated molecular patterns in the honeydew (VanDoorn et al. 2015).

### 5.3 Indirect Positive Effects of Honeydew on Plants

In addition to its direct positive effects, honeydew indirectly benefits plants in several ways. The presence of honeydew on plants helps attract beneficial insects, such as parasitoids and predators, that feed on it. Honeydew contributes to this in two ways: first, it directly serves as a food source for natural enemies due to its nutrient composition (Wäckers et al. 2008); second, the microorganisms in honeydew release volatiles with kairomonal properties that attract natural enemies (Leroy et al. 2011). For instance, bacterial volatiles from the cotton‐melon aphid, *Aphis gossypii* Glover honeydew have been shown to mediate oviposition site selection in ladybird beetles, a key predator of aphids. Volatiles such as DL‐lactic acid, 4,6‐dimethyl‐2‐heptanone, and didodecyl phthalate, produced by gammaproteobacteria *Acinetobacter* sp. and *Pseudomonas* sp., significantly attracted mated females of *Propylea japonica* and influenced their egg‐laying behavior (Li et al. 2025). This mechanism ensures that emerging larvae have immediate access to food sources, reinforcing natural aphid suppression and strengthening plant protection. This honeydew‐plant relationship benefits plants by promoting natural biological control, protecting them from insect herbivores (Figure 4) (Álvarez‐Pérez et al. 2023). Additionally, pollinator insects use honeydew as a source of carbohydrates, and while foraging for it, they visit numerous plants, thereby increasing the likelihood of plant pollination (Wäckers et al. 2008; Leroy et al. 2011).

### 5.4 Honeydew and Its Diverse Roles in Soil and Plant Ecosystems

**FIGURE 5:**
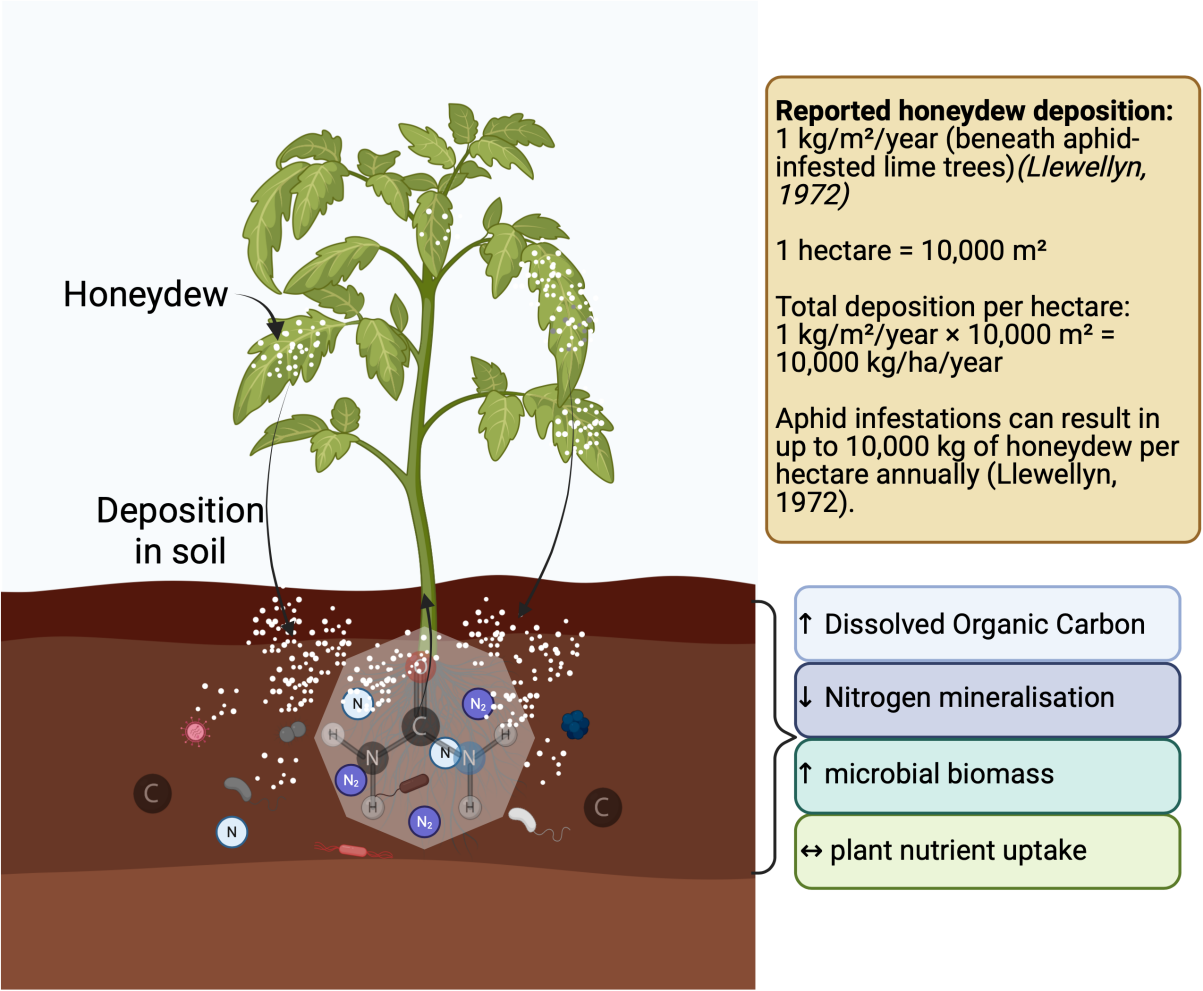
Aphid honeydew deposition modifies soil biogeochemistry by enhancing microbial biomass and DOC, while reducing nitrogen mineralization and plant‐available nitrogen, thereby influencing nutrient cycling and plant uptake.

Sap‐feeding insects excrete honeydew, which, though seemingly minute individually, has a substantial accumulative impact on a larger scale (Owen and Wiegert 1976; Buckley 1987). For example, aphids like *Tuberolachnus salignis* (Hemiptera: Aphididae) drain 1–4 mg of sugar daily from plants (Mittler 1958). Over its 30‐day life cycle, a single aphid can remove 30–120 mg of sugar. Even smaller aphids, such as *Eucallipterus tiliae* L., at about 1/20 the size of *T. salignis*, contribute by removing 0.38 mg of sugar daily (Llewellyn 1972; Llewellyn et al. 1974). Scaling up, a 14 m high lime tree may host over a million aphids at its peak, resulting in a daily release of 407 g of sugar per square meter and an estimated annual honeydew deposition of 1 kg m^−2^ yr.^−1^ beneath aphid‐infested lime trees (Figure 5) (Llewellyn 1972).

Honeydew produced by sap‐feeding insects such as aphids, scale insects, and leafhoppers, plays a crucial role in influencing soil fertility through various ecological interactions and nutrient dynamics. For instance, honeydew from scale insects significantly influences fungal community diversity and ecological interactions, serving as a vital carbon source supporting microbial and fungal activity in the soil (Dhami et al. 2011, 2013; Michalzik 2011).

Aphid‐produced honeydew, such as that secreted by *Cinara* spp., contributes to dissolved and particulate organic matter on the forest floor, enhancing soil nitrogen availability and net primary production. This contribution increases soil respiration and alters nitrogen fluxes, impacting overall soil fertility (Michalzik and Stadler 2005). In semi‐natural experiments under aphid‐infested and uninfested Norway spruce trees, solutions under infested trees showed higher concentrations of dissolved organic carbon (DOC) but lower concentrations of dissolved organic nitrogen (DON), NO_3_^−^‐N, and NH_4_^+^‐N in throughfall solutions, as well as lower NH_4_‐N in forest floor solutions (Michalzik and Stadler 2005). Interactions among *Tamarix* plants, the leafhopper *Opsius stactogalus* (Hemiptera: Cicadellidae), and litter fungi emphasize the broader ecological impact of insect‐produced honeydew on soil and plant health. Honeydew contributes organic matter and nutrients that support microbial biomass and activity, enhancing nutrient cycling processes crucial for maintaining soil fertility (Michalzik 2011).

Honeydew serves as an additional carbon source for soil microorganisms, particularly on the soil surface, leading to increased microbial biomass over time (Grier and Vogt 1990; Stadler and Michalzik 1998; Seeger and Filser 2008). This easily degradable carbon source stimulates microbial metabolism and activity, thereby enhancing soil fertility. Honeydew deposition affects throughfall composition, impacting soil solution chemistry and nutrient dynamics under Norway spruce trees (Stadler and Michalzik 1998). While honeydew does not directly increase available soil nitrogen, it may reduce nitrogen mineralization rates and nitrogen uptake by plants, negatively impacting soil fertility (Grier and Vogt 1990). Honeydew indirectly creates complex interactions within the soil food web. It influences not only microbial biomass but also the activity of soil fauna such as Collembola (springtails), which can indirectly benefit plant growth by enhancing soil health (Grier and Vogt 1990; Seeger and Filser 2008). Changes in soil chemistry due to honeydew deposition can influence plant nutrient uptake and overall plant fitness via the interconnectedness of aboveground aphid activity and belowground soil processes; honeydew impacts plant health through its effects on nutrient dynamics (Stadler and Michalzik 1998).

In conclusion, honeydew enriches microbial biomass and activity by providing a valuable carbon source, potentially enhancing soil fertility. It plays a significant role in influencing soil solution chemistry, thereby impacting nutrient dynamics. However, honeydew's influence on soil nitrogen availability may lead to a reduction, which could negatively affect soil fertility (Grier and Vogt 1990; Seeger and Filser 2008). Although honeydew does not directly boost plant fitness through increased soil nitrogen, it affects plant health through complex interactions within the soil ecosystem and changes in soil solution chemistry. These indirect effects can either promote or impede plant growth, depending on the dynamics within the soil environment (Grier and Vogt 1990; Stadler and Michalzik 1998; Seeger and Filser 2008). Collectively, these studies bring attention to the multifaceted role of honeydew in soil and plant ecosystems, emphasizing its dual impact on both soil fertility and plant fitness.

## 6 Agricultural Implications and Prospective Applications of Honeydew Insights

The impacts of honeydew on agriculture are significant, with both positive and negative effects. On the positive side, honeydew can support important ecosystem services like natural pest control and pollination (Álvarez‐Pérez et al. 2023; Ali et al. 2024; de Bobadilla et al. 2024). However, honeydew also has negative effects, such as reducing plant photosynthesis, lowering crop yields, and contaminating fruits, vegetables, and flowers, which makes them unfit for market (Capinera 2020; Ali 2023). Additionally, aphid honeydew increases microbial activity in the soil, altering nitrogen levels and potentially affecting plant nutrient uptake (Whitaker et al. 2014). Molds that develop on honeydew are also a significant threat, as they reduce photosynthesis and decrease crop yields. For example, Tosh and Brogan (2015) showed that untreated whitefly infestations, which cause honeydew deposition on plants, can lead to the growth of sooty mold. While sooty mold is often considered mainly an esthetic issue, its build‐up can reduce photosynthesis and negatively impact crop yields. In the United States, cotton growers suffer financial losses due to “sticky cotton” caused by sooty mold on cotton lint contaminated with aphid and whitefly honeydew (Hequet et al. 2007). From an ecological perspective, honeydew also plays a role in soil microbial processes, increasing microbial immobilization and potentially limiting nitrogen uptake by plants (Wardle 2013). Moreover, microorganisms alter the volatile properties and nutritional composition of honeydew, which can affect its interactions with the environment (Leroy et al. 2011; Francis et al. 2020; Liu et al. 2024). These diverse impacts of honeydew on agriculture highlight the importance of balancing its negative effects with its potential benefits for plant protection.

Beyond these known consequences, recent conceptual frameworks suggest potential pathways through which honeydew insights could be harnessed to support sustainable agricultural practices. For example, the documented specificity of honeydew volatiles provides a rationale for developing innovative monitoring tools such as biosensors or bioindicators to detect microbial signatures or volatile compounds in honeydew, offering real‐time information on pest presence or infestation intensity (Leroy et al. 2011). Additionally, the proven role of honeydew in recruiting natural enemies opens new opportunities for ecosystem service management, such as designing companion planting or intercropping systems to exploit these cues for enhanced biological control (Wäckers et al. 2008; Álvarez‐Pérez et al. 2023). Breeding programs could harness these insights by selecting cultivars with traits that alter honeydew composition or reduce its production, thereby minimizing pest attractiveness while maintaining defense mechanisms (Goggin 2007). Furthermore, understanding honeydew‐induced shifts in soil microbial communities could be explored as a means to support regenerative soil practices, particularly in optimizing nutrient cycling and reducing nitrogen immobilization (Stadler and Michalzik 1998; Michalzik 2011). While these strategies remain largely theoretical at present, they underscore the emerging potential of honeydew as a biological signal and management tool in agroecosystems. Future research should aim to test and validate these applications under field conditions to realize their practical benefits.

## 7 Conclusion and Future Prospects

Honeydew plays a multifaceted role in plant physiology, exerting both beneficial and detrimental effects. Negative impacts include the promotion of microbial pathogen proliferation, the development of sooty molds, and interference with photosynthesis, potentially reducing plant productivity. Conversely, honeydew can enhance plant defenses, regulate flowering, and contribute indirectly to soil fertility, supporting overall plant health. While this review synthesizes the current understanding of honeydew's dual role in plant physiology, it is important to acknowledge its limitations. Our analysis is constrained by the existing literature, which is notably uneven across different topics. For instance, the intriguing potential of honeydew to regulate flowering is based solely on studies from the 1970s, with no contemporary research to validate or explore these mechanisms in other plant systems. Furthermore, the heavy reliance on phenomenological observations in many studies means that the underlying molecular mechanisms and long‐term ecological consequences of honeydew deposition remain largely speculative. This review, therefore, highlights not only the state of the science but also the fragmented nature of the evidence, underscoring the need for more systematic and mechanistic investigations in the future. Future research should explore the mechanisms underlying honeydew‐mediated plant diseases, including the spread of secondary infections and their impact on overall plant fitness. Additionally, studying the effects of honeydew deposition on photosynthesis and its long‐term consequences for plant growth and productivity is essential. Furthermore, it is important to consider honeydew in agricultural practices, recognizing that its impacts extend beyond farming and affect various aspects of ecosystem functioning. Effective management strategies are crucial to reducing the negative effects of honeydew on crop yields and overall agricultural productivity. By addressing these research gaps and implementing comprehensive management approaches, it will be possible to use honeydew to optimize plant health and promote ecosystem resilience.

## Author Contributions

J.A. R.C. A.B. A.A.Y., and E.C. conceived the idea. J.A., C.R., M.D., R.R. T.D. A.A.Y., E.C., and A.B. wrote the first draft of the paper with input from all authors.

## Conflicts of Interest

The authors declare no conflicts of interest.

## Data Availability

This review paper does not contain original data; therefore, data availability is not applicable.
